# The chemical biogeography of a widespread aromatic plant species shows both spatial and temporal variation

**DOI:** 10.1002/ece3.9265

**Published:** 2022-09-09

**Authors:** Ken Keefover‐Ring

**Affiliations:** ^1^ Department of Botany University of Wisconsin‐Madison Madison Wisconsin USA; ^2^ Department of Geography University of Wisconsin‐Madison Madison Wisconsin USA

**Keywords:** carvacrol, chemical biogeography, essential oils, geraniol, linalool, *Monarda fistulosa*, terpenoids, thymol

## Abstract

Plants produce a wide variety of secondary metabolites, but intraspecific variation in space and time can alter the ecological interactions these compounds mediate. The aim of this work was to document the spatial and temporal chemical biogeography of *Monarda fistulosa*. I collected leaves from 1587 *M. fistulosa* individuals from 86 populations from Colorado to Manitoba, extracted and analyzed their terpenes with gas chromatography, mapped monoterpene chemotypes, and analyzed chemical variation with principal component analysis. I also measured the amounts of terpenes in different plant tissues to examine intraplant variation and monitored leaf terpene chemistry over a single growing season to examine temporal patterns. Finally, I extracted terpenes from herbarium samples up to 125 years old and compared the chemotypes with recent samples from the same sites. *M. fistulosa* populations consisted mostly of thymol (T) and carvacrol (C) chemotypes, but geraniol (G) and (*R*)‐(−)‐linalool (L), a chemotype new to this species, were also present. A principal component analysis showed that most of the chemical variation across populations was due to the amounts of the dominant terpene in plants. Intraplant tissue chemistry revealed that leaves mostly had the greatest amounts of terpenes, followed by floral structures, stems, and roots. Short‐term temporal variation in leaf chemistry of T and C plants over a growing season showed that plants produced the highest levels of biosynthetic precursors early in the season and their dominant monoterpenes peaked in mid‐summer. Plant chemotype was discernable in the oldest herbarium samples, and 15 of 18 historic samples matched the majority chemotype currently at the site, indicating that population chemotype ratios may remain stable over longer time scales. Overall, the results show that plant species' secondary chemistry can vary both spatially and temporally, which may alter the biological interactions that these compounds mediate over space and time.

## Graphical abstract

Plants produce a wide variety of secondary metabolites, but intraspecific variation in space and time could alter the interactions these compounds mediate. The aim of this work was to document the spatial and temporal chemical biogeography of *Monarda fistulosa*. Overall, the results show that plant species’ secondary chemistry can vary both spatially and temporally, which may alter the biological interactions that these compounds mediate over space and time.

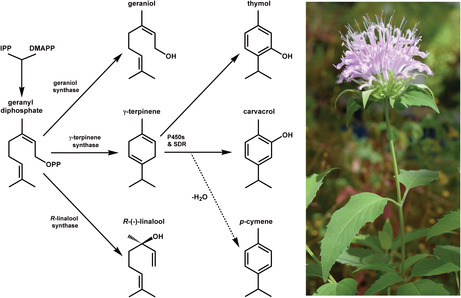

## 1 INTRODUCTION

Plants are heterogeneous resources for the organisms with which they interact and one major source of plant heterogeneity is variation in the amounts and composition of the secondary metabolites that they produce. As the name implies, secondary metabolites are not part of primary plant metabolism (e.g., sugars, proteins, nucleic acids) but are specialized compounds (e.g., alkaloids, terpenoids), often with high taxonomic affinity. Variation in plant secondary chemistry can occur at many scales, both spatially and temporally. Spatial variation in secondary metabolites can occur at the scale of a single individual with differences among different plant tissues (Bowers & Stamp, 1992), among individuals within populations (Keefover‐Ring et al., 2009), and among different populations, up to regional and large geographic scales (Keefover‐Ring et al., 2014; Pratt et al., 2014). Temporal variation can happen over rapid time scales, such as induction (Karban & Baldwin, 1997; Keefover‐Ring et al., 2016), with plant ontogeny (Barton & Koricheva, 2010), or over longer evolutionary time scales, as the selective forces acting on plant populations change over time (Thompson et al., 2013).

From an ecological point of view, understanding the spatial and temporal variation of the secondary chemistry of a species is important since these compounds are involved in many biological interactions between the plants that make them and other species. The interactions that these chemicals mediate can be either mutually beneficial, such as the attraction of pollinators, or antagonistic, such as the deterrence of herbivores and allelopathy (Gershenzon & Dudareva, 2007; Langenheim, 1994). Furthermore, these interactions often operate bi‐directionally, such that the organisms surrounding a plant can also affect the chemistry of the plant population (Linhart, 1991). In addition to biotic forces, a plant's secondary chemical composition can also be shaped by abiotic forces, such as nutrients (Loney et al., 2006), moisture availability (Johnson et al., 1997), or temperature (Pratt et al., 2014; Thompson et al., 2007), and these forces can vary over the landscape and with time, resulting in different secondary chemistry‐driven ecological and evolutionary outcomes (Thompson, 2005). However, large‐scale studies to determine the phytochemical landscape (Hunter, 2016) of a species can be time‐consuming and costly and have only been done for a limited number of species (e.g., Bohm, 2009; Gouyon et al., 1986; Keefover‐Ring et al., 2014).

The focus of this work is to document the spatial and temporal chemical biogeography of one plant species, *Monarda fistulosa* L. var. *menthifolia*, in at least part of its extensive range. Like many species in the Lamiaceae, *M. fistulosa* synthesizes essential oils (a mixture of mono‐ and sesquiterpenes) in mostly peltate granular trichomes on the surfaces of flower petals, calyces, bracts, leaves, and even stems (Heinrich, 1973; Pfab et al., 1980). The pattern of essential oil production in *M. fistulosa* is a chemical polymorphism where individuals are identified with distinct chemical phenotypes, or chemotypes, where a single monoterpene dominates (Keefover‐Ring et al., 2009), which is consistent with other labiate species (Fleisher & Sneer, 1982; Vernet et al., 1986). Except for the work of Marshall and Scora (1972) and Keefover‐Ring (2015), however, all other studies documenting *M. fistulosa* chemistry analyzed relatively few individuals.

The largest spatial scale in this study includes mapping the chemotypes of *M. fistulosa* populations from southern Colorado and extending north to sites in Wyoming, the Dakotas, and Manitoba, which included collecting and analyzing 1587 individuals from 86 separate populations. On a much smaller spatial scale, the amounts of terpenes in different plant tissues were measured to examine intraplant variation. One temporal component of the work dealt with measuring phenological changes in leaf terpene chemistry of *M. fistulosa* plants of the two dominant chemotypes over a single growing season and into senescence. A longer‐term perspective was taken to see whether the chemotypes of particular populations have changed over time by comparing chemical analyses from recent sample collections to the terpenes extracted from herbarium samples collected up to 125 years ago from the same sites.

The specific questions addressed in this study were: (1) What is the chemotype distribution of *M. fistulosa* populations in at least part of its extensive range? (2) What is the composition and abundance of terpenes in different *M. fistulosa* tissues? (3) How do terpenes vary over the growing season? and (4) Is plant chemotype detectable from historic herbarium specimens and do they match the dominant contemporary chemotype in the same populations?

## 2 MATERIALS AND METHODS

### 2.1 Study organism

*Monarda fistulosa* var. *menthifolia* (Graham) Fernald (Lamiaceae; hereafter *Monarda fistulosa*), a member of the subgenus *Monarda* (Prather et al., 2002), commonly known as wild bergamot, bee balm, or horse mint, is a perennial mint that occurs in all of the continental United States, except for Alaska, California, Florida, and all of the southern Canadian provinces, as far east as Quebec, and in the Northwest Territory (Straley, 1986; USDA, 2008).

*M. fistulosa* hosts a variety of herbivores, including a seed‐eating pyralid (Davis et al., 1988) that substantially reduces seed set, the specialist aphid *Aphis monardae* (Wyckhuys et al., 2007), and the monophagous one‐spotted tortoise beetle *Physonota unipunctata* (Criddle, 1926; Hamilton, 1884; Sanderson, 1948). The larvae of these tortoise beetles protect themselves by concentrating plant terpenes in “fecal shields” (Keefover‐Ring, 2013) and while they drastically reduced plant fitness of both of the chemotypes tested, larval performance differed between chemotypes (Keefover‐Ring, 2015).

**FIGURE 1:**
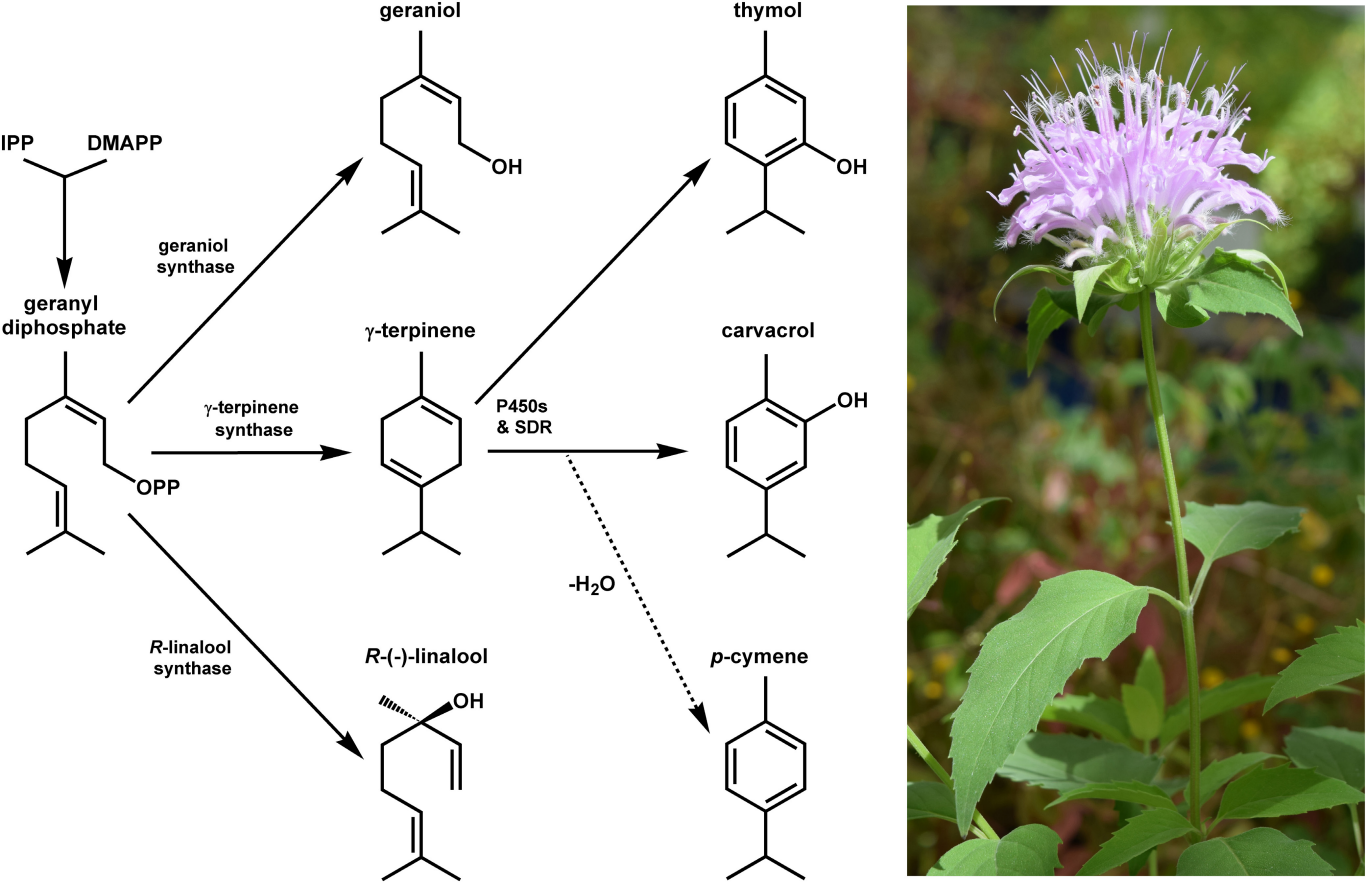
Biosynthesis of the major monoterpenes found in the essential oil of *Monarda fistulosa* in this study. The precursor's isopentenyl pyrophosphate (IPP) and dimethylallyl pyrophosphate (DMAPP) lead to geranyl diphosphate (GPP), which is then converted to geraniol, γ‐terpinene, or (*R*)‐(−)‐linalool. Various cytochrome P450s and a short‐chain dehydrogenase (SDR) convert γ‐terpinene to thymol and carvacrol (Krause et al., 2021). *p*‐Cymene results from the dehydration of a carvacrol intermediate (Krause et al., 2021). Photo: Ken Keefover‐Ring.

Previous research identified three chemotypes of *M. fistulosa*, defined by the presence of a predominant amount of one of three different monoterpenes: thymol (T; scent of thyme), carvacrol (C; scent of oregano), or geraniol (G; lemon‐like scent; Johnson et al., 1998; Keefover‐Ring, 2013, 2015; Marshall & Scora, 1972; Scora, 1967; Weaver et al., 1995; Figure 1). Unlike many other monoterpenes that are aliphatic in character, thymol and carvacrol are based on a phenolic structure and are only produced by a few other labiate species, including species in the genera of *Thymus* and *Oregano* (Chizzola, 2003; Fleisher & Sneer, 1982; Skoula & Grayer, 2005; Stahl‐Biskup & Saez, 2002), and other species in the genus *Monarda* (Burt, 1936; Scora, 1967). Plants that produce thymol and carvacrol almost always also have relatively high amounts of the aliphatic monoterpenes γ‐terpinene and *p*‐cymene. γ‐Terpinene serves as the biosynthetic precursor for thymol and carvacrol and *p*‐cymene results from dehydration of a dienol intermediate that leads to carvacrol (Krause et al., 2021; Figure 1).

### 2.2 Chemical biogeography of *M. fistulosa*

#### 2.2.1 Sample collection and preparation

To investigate geographic variation in *M. fistulosa* essential oil chemistry, I collected single leaves from 1587 individual plants at 86 different populations for terpenoid analysis from 2002 to 2007 (Table A1). The majority of the populations sampled were located in Colorado (66 sites) and specifically in Boulder County (41 sites). There were also 14 sites in southern Manitoba, Canada, three in North Dakota, two in South Dakota, and one in Wyoming. The number of individuals per site ranged from one to 122 with a mean of 18 (*SD* = 14.2). Sites were chosen using collection records from the University of Colorado Museum Herbarium (COLO) and by observing populations, usually in flowers, when driving or hiking. At each site, plants were haphazardly chosen with enough distance between them to ensure that the stems sampled were from different individuals. Typically, the first leaf immediately below the bracts was detached next to the stem, rolled to fit into a 2 ml microcentrifuge tube, and completely submerged with either 1.00 or 1.50 ml of ethanol, containing *m*‐xylene as an internal standard (0.1 μl/ml). Usually, the entire procedure was carried out in the field within a few hours of collection, or when not possible within 24 h of collection, during which time leaf samples were stored in either a small cooler with ice or a refrigerator. Upon return to the lab, samples were placed in a sonication bath for 15 min and then allowed to extract for 7 days at room temperature. After the 7‐day soaking period, 100 μl of the solution from each sample was combined with 100 μl of internal standard solution and injected into a gas chromatograph (GC). Leaves were later removed from the solvent, dried to a constant weight at 70°C, and weighed to the nearest mg.

#### 2.2.2 Chemical analysis

I measured the concentrations of 14 different compounds known to be the major components of *M. fistulosa* essential oil (Keefover‐Ring, 2013, 2015; Marshall & Scora, 1972; Scora, 1967; Weaver et al., 1995). The monoterpenes assayed included myrcene, limonene, γ‐terpinene, *p*‐cymene, *cis*‐sabinene hydrate, linalool, carvacrol methyl ether, nerol, geranyl acetate, geraniol, thymol, and carvacrol. The levels of a single sesquiterpene, germacrene D, and the alcohol octen‐3‐ol were also measured. All plant sample compound identification and quantification were carried out with GC with the methods used in Keefover‐Ring et al. (2009), and final amounts were reported in mg compound per gram of plant tissue dry weight (mg/g DW). I performed additional analyses to determine the ratio of the two enantiomers of linalool, a chiral compound, using methods from Keefover‐Ring et al. (2016). I also converted the concentration data of all compounds in the samples to percentages and assigned each plant a chemotype based on the compound with the largest percentage of the total (Keefover‐Ring et al., 2009).

#### 2.2.3 Statistical analysis

All statistical analyses in this study were carried out using SAS version 9.4 (SAS Institute Inc, 2013). In addition to mapping the spatial patterns of the essential oil chemotypes using ArcMap (Esri), I further characterized *M. fistulosa* chemistry over the landscape with a principal components analysis (PCA; PROC PRINCOMP in SAS) using the concentrations (mg/g DW) of all 14 measured compounds as the dependent variables. The PCA included chemical analysis results for 1007 plants [T (*N* = 419), C (*N* = 568), G (*N* = 17), and L (*N* = 3)] from 54 sites. Plant chemotype was determined for all 1587 samples in the chemotype mapping part of the study using dominant compound peak areas, however, the leaf dry weights needed to calculate compound concentrations were only available for 1007 samples. Thirty‐nine of these sites were from Colorado (including the L chemotype site), 14 from Manitoba (including all sites containing G chemotype plants), three from North Dakota, two from South Dakota, and one from Wyoming.

### 2.3 Intraplant chemical variation

#### 2.3.1 Sample collection and preparation

To assess the essential oil chemistry of various tissues (intraplant) of *M. fistulosa*, single stems from 13 separate C chemotype plants, including some roots, were collected from the Crescent Meadows (Table A1) population in Colorado on July 18, 2003. The stems were dissected and various parts were soaked separately in 1.00 ml internal standard solution in 2 ml microcentrifuge tubes. Separate parts included flowers (including petals and all sexual parts), calyces, the bracts that subtend the inflorescence, leaves from the first, second, and third positions below the bracts, stems, and roots. This process was repeated on July 28, 2004, with single stems from five separate geraniol chemotype plants from Spruce Woods Provincial Park in Manitoba. This time the parts used included only flowers, calyces, bracts, only the second leaf below the bract, and stems. All intraplant samples were analyzed by GC, as above.

#### 2.3.2 Statistical analysis

The proc GLM function was used to test for differences in γ‐terpinene, *p*‐cymene, thymol, carvacrol, and total terpenes between the various parts of the 13 C chemotype plants. Compounds with significant ANOVAs were then subjected to pair‐wise comparisons with a Ryan‐Einot‐Gabriel‐Welsch multiple range test. Since carvacrol methyl ether is usually either present or absent in individuals, only the three plants in which it occurred were used in the statistical analysis of this compound. The same statistical procedure was used to look for chemical differences among parts of the five G chemotype plants. In this case, only the main monoterpene geraniol and total terpenes were examined. To meet ANOVA assumptions of normality individual terpene data were square root transformed.

### 2.4 Seasonal ontogeny of monoterpenes

#### 2.4.1 Sample collection and preparation

To determine the changes in levels of terpenes that occur over an entire season, 14 T and 16 C chemotype plants were identified from a separate experiment located on Flagstaff Mountain in the foothills ~2 km west of Boulder, CO (Keefover‐Ring, 2015). During the 2007 growing season, leaf collections were made from all 30 individuals on five separate occasions. On April 19, when most plants had only vegetative shoots protruding about 5 cm from ground level, one or more terminal leaves were collected from a single stem of each plant. Subsequent samples consisted of the first leaf below the reproductive parts and were collected on June 1 when most plants had small terminal flower buds, on July 19 when most were in full flower, and on September 28 when plants had no flowers but still mostly green foliage. The final collection was on December 3. At this time plants still had many of their leaves, but almost all foliage had become dry and brown. All samples were soaked in 1.00 ml internal standard solution and analyzed by GC, as above.

#### 2.4.2 Statistical analysis

I performed individual repeated measures analyses for γ‐terpinene, *p*‐cymene, thymol, carvacrol, and total terpenes separately for the two different chemotypes for the five different time periods using PROC MIXED. Compounds that were significant for the repeated factor of time were then subjected to pair‐wise comparisons with a Ryan‐Einot‐Gabriel‐Welsch multiple range test. To meet ANOVA assumptions of normality individual terpene data were transformed as above.

### 2.5 Historic samples

#### 2.5.1 Sample collection and preparation

To evaluate whether the chemotypes of historic herbarium samples of *M. fistulosa* could be determined for comparison to present populations, I analyzed 44 separate accessions of *M. fistulosa* from the University of Colorado Museum Herbarium (COLO; Table A2) by GC‐mass spectrometry (MS). Original collection dates ranged from 1872 to 2001, with at least one sample from every decade during that time, except for 1890. The accuracy of location information varied among the accessions, with generally more specific location placement for the more recent samples. However, even some of the earliest sites sampled were well‐described.

Samples for chemical analysis consisted of approximately one‐half of a leaf (usually the first leaf below the bracts) cut from each pressed specimen and placed in separate small manila envelopes. Eight to twelve milligrams of the dried leaf material was weighed to 0.1 mg, placed in small glass vials with PTFE‐lined screw tops, and 0.50 ml of an internal standard solution (*m*‐xylene in *n*‐hexane, GC^2^ hexane, Burdick and Jackson) was added and samples sonicated for 15 min. Samples were allowed to soak for 7 days before analysis.

Twenty of the historic sites were also locations where contemporary plants were collected for the chemical biogeography work described above (Table A2). Many of the remaining historic sites were revisited, but either the exact location of the population could not be found or no current *M. fistulosa* population still existed.

#### 2.5.2 Chemical analysis

Historic samples were analyzed with an Agilent 6890 N GC/Agilent 5975 MS with an HP‐1MS column (30 m × 0.25 mm I.D., film thickness 0.25 μm, Agilent Technologies, Inc.). One microlitre of each sample was injected in the splitless mode with oven conditions that included an isothermal hold at 60°C for 5 min, followed by a ramp of 10°C/min to 250°C. Linear retention indices were also calculated on the HP‐1 column with the same oven conditions used when determining them on the DB‐Wax column.

#### 2.5.3 Statistical analysis

I used a χ^2^ statistic to test whether the observed chemotypes from herbarium samples differed from the chemotypes expected based on the majority of chemotype detected at the sites from the recent sampling. Also, while many of the historical samples contained very small amounts of several different terpenes, only *p*‐cymene, thymoquinone, carvacrol methyl ether, thymol, and carvacrol were present in relatively large amounts in most of the samples and were the only plant terpenes used for statistical analyses. Using the PROC REG function, the concentration data for *p*‐cymene, thymoquinone, carvacrol methyl ether, a total of the two phenolic monoterpenes (thymol and carvacrol), and a total of all five were regressed on the sample collection date.

## 3 RESULTS

### 3.1 Chemical biogeography of *M. fistulosa*

**FIGURE 2:**
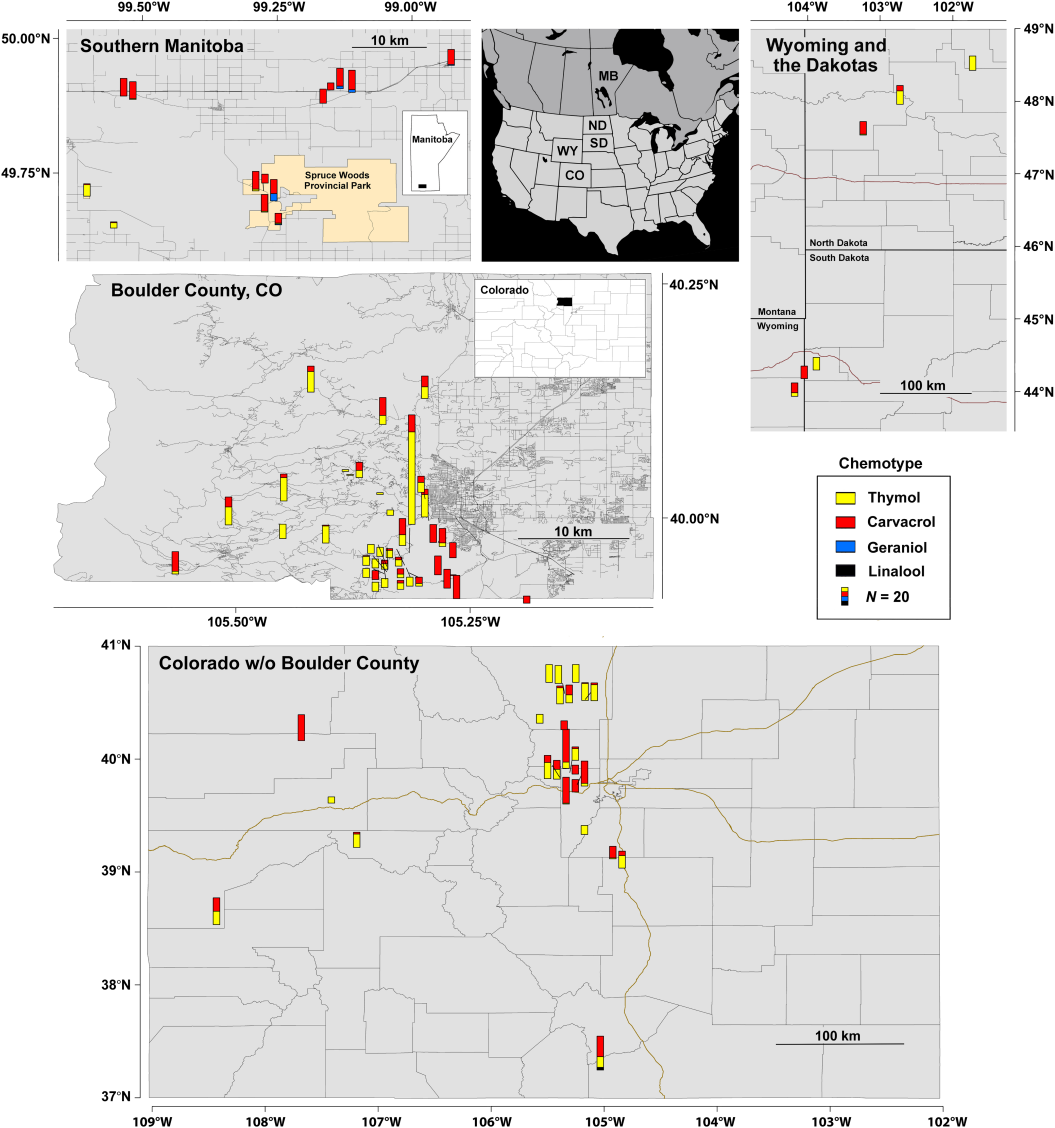
Chemotype mapping of *Monarda fistulosa* populations in southern Manitoba, Wyoming, North and South Dakota, Colorado (excluding Boulder County), and Boulder County, CO. yellow = thymol (T), red = carvacrol (C), blue = geraniol (G; southern Manitoba), and black = (*R*)‐(−)‐linalool (L; Colorado site farthest south) chemotype plants. The bottom right of each bar is the actual site location. When present, leader lines point to site location. Map of North America shows the US states, Colorado (CO), Wyoming (WY), South Dakota (SD) and North Dakota (ND), and the Canadian province of Manitoba (MB) where populations were sampled. Inset in the southern Manitoba map shows the location of that site in the province, and inset in the Boulder County map shows its location in Colorado.

Four different chemotypes of *M. fistulosa* were found as a result of sampling 1587 individual plants for this study, with either geraniol (G), linalool (L), thymol (T), or carvacrol (C) as an individual plant's dominant monoterpene (Figure 2). Plants of the T (*N* = 774 plants) and C (*N* = 793) chemotypes were the most common and either one or both were found at all of the 86 sites examined. The two other chemotypes, G (*N* = 17) and L (*N* = 3), comprised only a small percentage of the total. Thirty‐eight sites were monomorphic for a single chemotype with 17 containing only C plants and 21 only T. Only two sites had more than two chemotypes present, including one site in southern Colorado (Highway 12), which had T, C, and L plants and a single site in southern Manitoba, which had T and C chemotypes together with G plants.

#### 3.1.1 Colorado

The majority of the populations sampled were located in Colorado (66 sites) and specifically in Boulder County (41 sites), ranging in elevation from 1797 m (Rocky Flats) to 2694 m (Eldora). Most populations were in the foothills in fairly mesic clearings, usually in ponderosa pine forests. In addition to the two common T and C chemotypes, three individual plants of a new chemotype with linalool (L) as the main component of their essential oil, were found in the southern‐most Colorado population. This represents a chemotype previously unknown for *M. fistulosa*. Analysis with a chiral GC column revealed a dominant amount of the (*R*)‐(−)‐linalool enantiomer (scent of lavender; $\bar{x}$ = 99.4%, SD = 0.1) in all three plants.

#### 3.1.2 Wyoming and the Dakotas

Six populations were sampled in Wyoming and the Dakotas. Three semi‐natural prairie sites were in northwestern North Dakota with two northern sites dominated by T chemotype plants and the more southern location containing mostly C plants. The two populations in South Dakota and the Wyoming site were in the Black Hills in mostly ponderosa pine forest. The northernmost of these three sites were monomorphic for T plants, while the other two had either all or almost all C chemotype plants.

#### 3.1.3 Manitoba

Plants from the 14 Manitoba populations consisted of T, C, and G chemotypes. Five of the populations were within Spruce Woods Provincial Park and the remaining seven were from areas surrounding the park. Twelve populations were dominated by C chemotype plants, including four that were monomorphic for C. Two populations contained T as the dominant chemotype, but both had one C plant present. G chemotype plants were detected at five sites and except for one location that had a single T and G plant, always co‐occurred only with C plants. G plants were usually at low densities; however, at one site they accounted for more than a third of the plants analyzed.

#### 3.1.4 Principal component analysis of *Monarda fistulosa* leaf compound data from field collections

**FIGURE 3:**
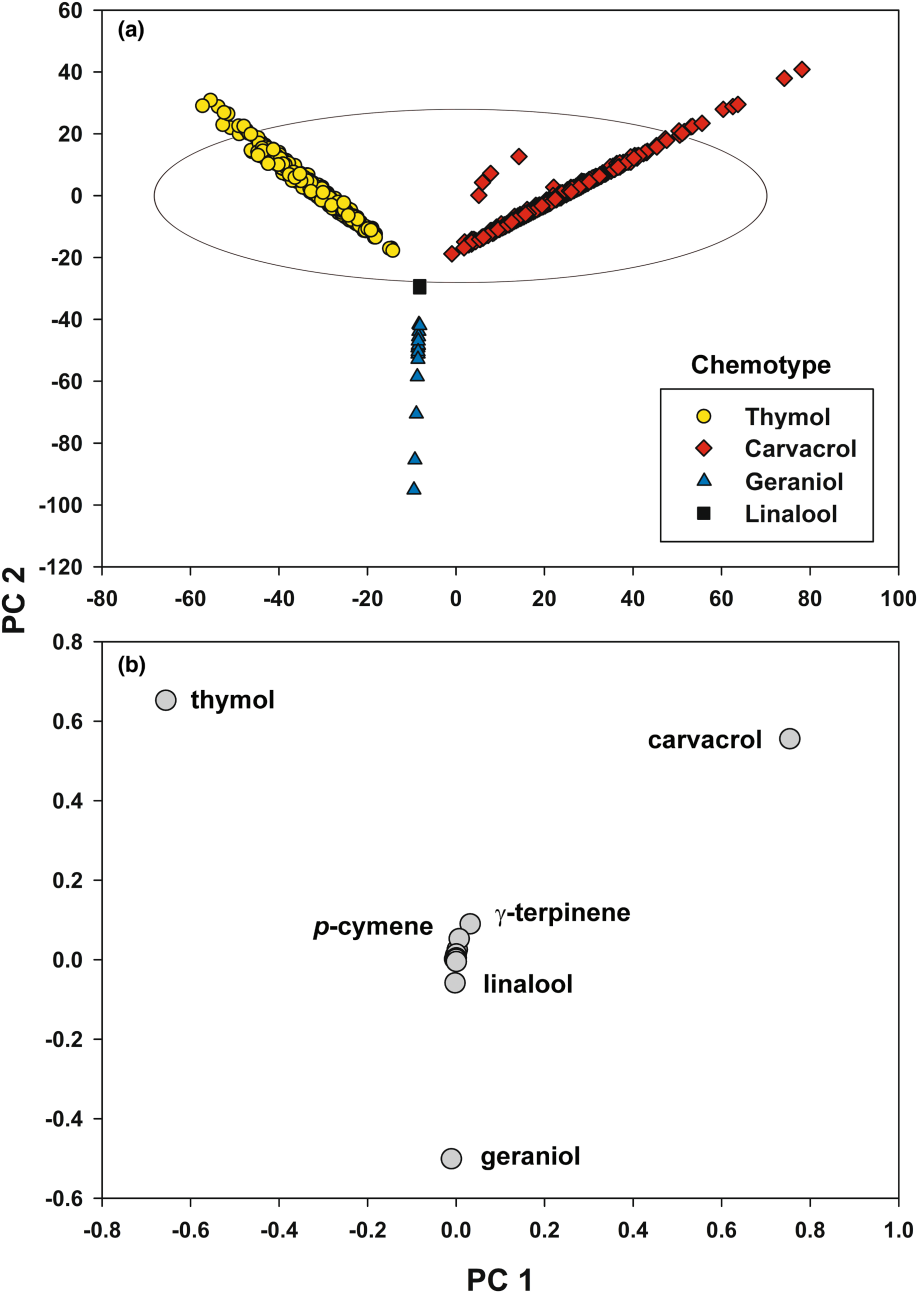
Principal component analysis (PCA) of concentration data of 14 compounds from 1007 *Monarda fistulosa* individuals from 54 populations from southern Colorado to southern Manitoba (a) the first two principal components showing the four terpene chemotypes (thymol carvacrol, geraniol, and (*R*)‐(−)‐linalool) with the 95% prediction ellipse shown. (b) A plot of the eigenvectors of the 14 compounds used for the PCA with key terpenes labeled.

The first two principal components (PC) using the concentration data of 14 compounds from 1007 *M. fistulosa* individuals over 54 populations from southern Colorado to southern Manitoba explained 91.0% of the variation (79.1% and 11.9% for PC 1 and PC 2, respectively; Figure 3a). PC 1 mostly represented the concentrations of thymol and carvacrol in respective T and C chemotypes. These two chemotypes had very similar amounts and percentages of the total essential oil of their main monoterpene (Table 1). Plants with increasing amounts of thymol (concentration, not percentage) corresponded to increasingly negative PC 1 values. C chemotype individuals showed a similar relationship, but higher positive PC 1 values were correlated with greater carvacrol amounts. In addition to variation in thymol and carvacrol amounts, PC 2 also included the variation in geraniol concentrations of the G chemotype plants from Manitoba. The three L plants were also a small part of the variation of PC 2. G and L plants both had higher amounts of their main monoterpene than T and C plants and a much higher percentage of the total essential oil (Table 1). While the component scores of some plants within sites were grouped closely together (see Data Availability Statement), the overall pattern was a result of variation in concentration of the main terpene in the various chemotypes.

**TABLE 1:** Means, standard deviations (SD), and ranges of the content (mg/g) of the four dominant monoterpenes in the four *Monarda fistulosa* chemotypes and their percentages (%) of the total essential oil

Main chemotype terpene	Thymol *N* = 419	Carvacrol *N* = 568	Geraniol *N* = 17	Linalool *N* = 3
Content [mg/g (SD)]	38.8 (15.7)	42.5 (14.1)	61.4 (30.1)	85.9 (6.3)
Range	9.8–79.1	9.4–114.7	34.6–141.9	81.0–93.0
% of total essential oil (SD)	74.7 (5.4)	75.1 (7.5)	95.8 (0.7)	97.5 (0.2)
Range	42.6–83.3	37.7–89.0	94.4–96.7	97.3–97.6

A plot of the eigenvectors of the 14 compounds used for the PCA (Figure 3b) explained why the arrangement of the component scores of *M. fistulosa* individuals (Figure 3a) was mostly due to the concentrations of the main terpenes in T, C, and G chemotype plants. The eigenvectors for thymol and carvacrol were widely spaced across the upper quadrants of PC 1 and both relatively far from the zero point of PC 1. Thymol and carvacrol eigenvectors also deviated considerably from the PC 2 zero point, but geraniol did as well (negative PC 2 eigenvector values). The remaining compounds grouped closely in the center of the eigenvector plot and contributed very little to the component score pattern.

### 3.2 Intraplant chemical variation

**FIGURE 4:**
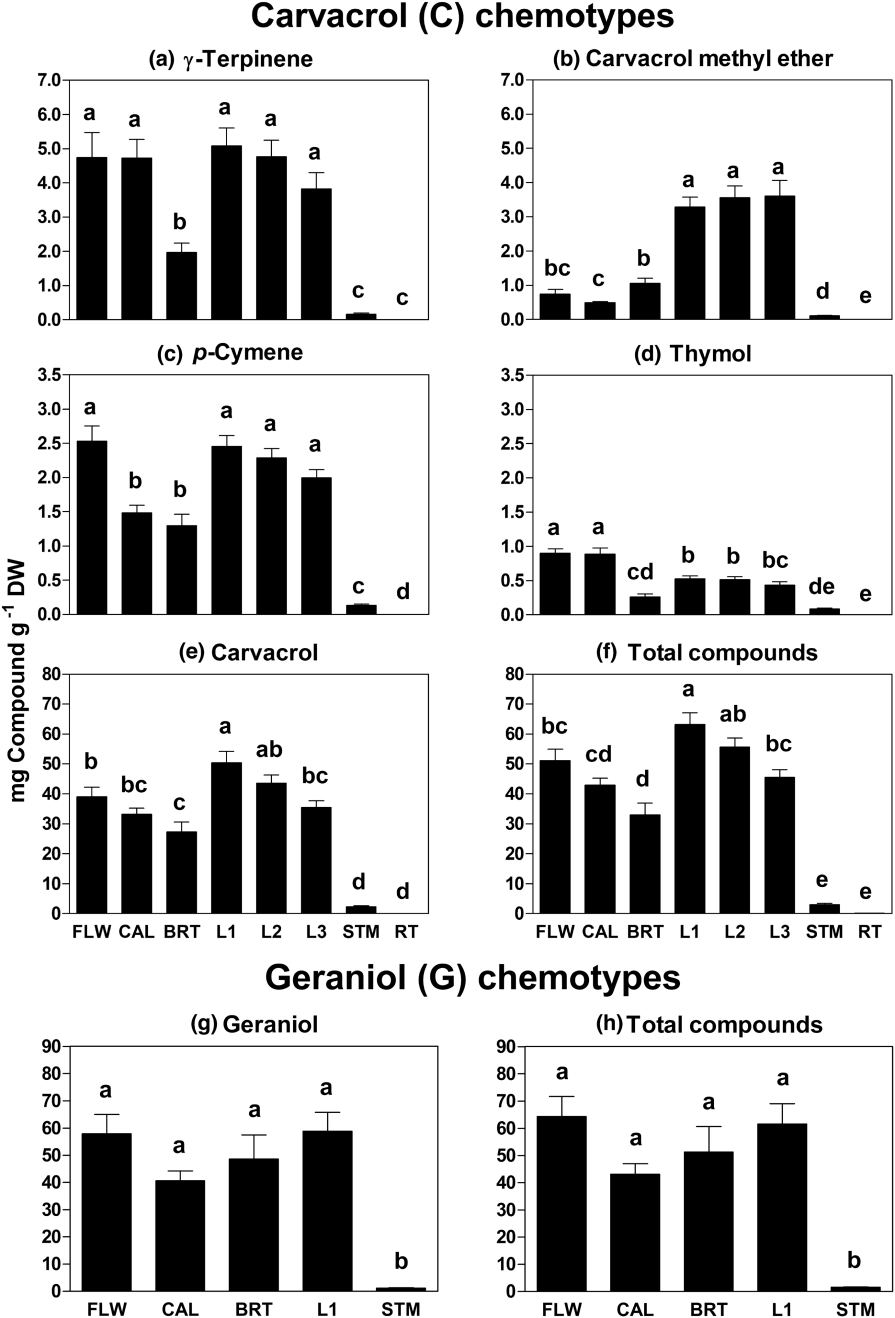
Intraplant terpene variation in *Monarda fistulosa*. Mean content [mg/g dry weight (DW) ± SE] of the main terpenes and total compounds in different tissues of 13 carvacrol (C) chemotype (a–f) and five geraniol (G) chemotype plants (g and h). FLW = corollas (including sexual parts); CAL = calyces; BRT = bracts; L1–L3 = first, second, and third leaves below the capitula; STM = stem; RT = root. Root samples contained terpenes, but in levels too low to be seen in the plots. Leaves L1 and L3 and roots were not collected from G plants. Significant ANOVA tests (α = 0.05) were followed by post hoc testing, indicated by letters above the bars. Means with the same letters were not significantly different. Note the difference in scales.

Different *M. fistulosa* plant tissues varied in their content and composition of terpenes (Figure 4). In most cases, stems and roots differed from all other parts and had the lowest concentration of essential oils. In the 13 C chemotype plants analyzed, γ‐terpinene differed between the various tissues (*F*_7,96_ = 59.84, *p* < .001) and was found at its highest levels in flowers, calyces, and leaves, all of which differed from bracts, stems, and roots (Figure 4a). The lowest amount of this compound was in stems and roots, which differed from bracts. *M*. *fistulosa* parts had different carvacrol methyl ether contents (*F*_7,16_ = 98.48, *p* < .001), including low levels in flowers, calyces, and bracts compared with leaves (Figure 4b). *p*‐Cymene content also differed between tissues (*F*_7,96_ = 66.98, *p* < .001) in a pattern similar to that of γ‐terpinene, except for much lower amounts in calyces and roots (Figure 4c). The different tissues varied in their thymol content (*F*_7,96_ = 43.94, *p* < .001), the minor phenolic in these C plants, with the highest amount in flowers and calyces, which differed from all other tissues (Figure 4d). Levels of the dominant monoterpene carvacrol and total terpenes also varied between tissues (*F*_7,96_ = 51.37, *p* < .001 and *F*_7,96_ = 66.81, *p* < .001, respectively) with the highest levels in the first leaf and then decreasing with leaf position and from flowers to bracts (Figure 4e,f).

The trends for essential oils among the parts of G chemotype plants were similar to those of C plants, but the only statistical differences were between the low amounts seen in stems compared with all other tissues (Figure 4g,h). The main monoterpene geraniol, which constituted most of the essential oil (Figure 4g), and the total compounds (Figure 4h) both differed between tissues (*F*_4,20_ = 14.88, *p* < .001 and *F*_4,20_ = 15.07, *p* < .001, respectively) and in a similar pattern.

### 3.3 Seasonal ontogeny of monoterpenes

**FIGURE 5:**
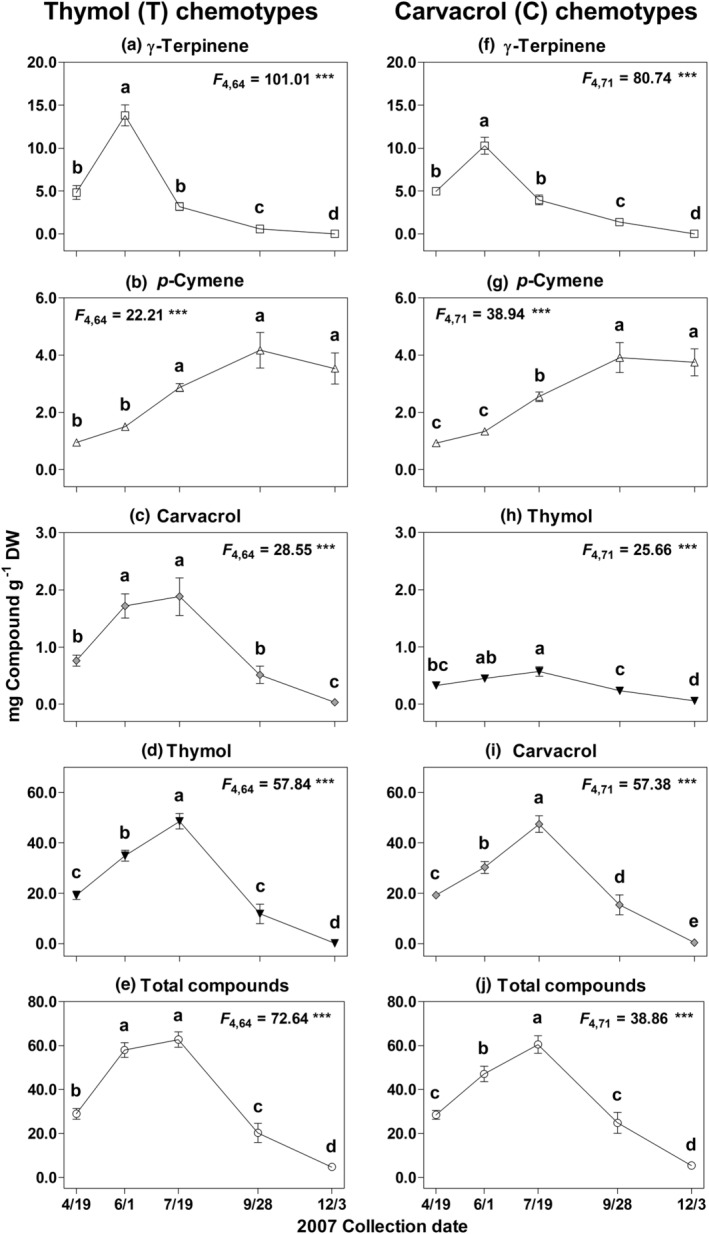
Ontogenetic changes of the main terpenes and the total in *Monarda fistulosa* foliage over a growing season. Mean (± SE) amounts in mg terpene g^−1^ dry weight (DW) of the main terpenes and total compounds in 14 thymol (T) and 16 carvacrol (C) chemotype plants at five time points during the 2007 season. *F* and significance values (***p < .001) are for the repeated measures analyses factor of time. Letters above points indicate results of post hoc testing. Means with the same letters were not significantly different. Note the difference in scales.

Plants of both T (Figure 5a–e) and C (Figure 5f–j) chemotypes, followed over the 2007 growing season, displayed differences in the amounts of all compounds between at least two of the five collection periods [all significant (*p* < .001) for the repeated measures factor of time]. In both T (Figure 5a) and C (Figure 5f) chemotype plants, the levels of γ‐terpinene showed no differences among the April and July collection dates, but were higher in June, lower at the end of September, and by December could no longer be detected in leaves. Levels of *p*‐cymene (Figure 5b,g) were their lowest in April and June, increased to their highest amount in late September, and remained there for the rest of the year. Amounts of the dominant monoterpene in both chemotypes [Figure 5d (thymol) and 5i (carvacrol)] increased steadily from April, peaked in July, and then dropped to very low levels by early December. In addition, there was always more of the minor phenolic carvacrol in T plants (Figure 5c) than thymol in C plants (Figure 5h). Total compounds mostly mirrored the patterns seen for the dominant terpenes (Figure 5e,j), except for relatively high total amounts in T plants in June, due to greater levels of γ‐terpinene and *p*‐cymene.

### 3.4 Comparing chemotypes of historic and contemporary sites

All of the historic *M. fistulosa* herbarium samples contained measurable amounts of terpenes and plant chemotype was able to be determined for all, except for the sample from Boulder Canyon, which had equal amounts of carvacrol and thymol. Besides *p*‐cymene, thymoquinone, carvacrol methyl ether, thymol, and carvacrol, some of the historic samples also contained small amounts of compounds found in contemporary samples, including α‐pinene, limonene, and octen‐3‐ol. One sample from Palmer Lake, CO, originally collected in 1901, contained a large amount of geraniol, but this monoterpene was not seen in any appreciable amount in samples collected at that site in 2003, or in any other plants collected in Colorado.

The contemporary resampling of 20 populations where herbarium samples had been collected yielded nine sites where T chemotypes dominated and ten sites where C chemotypes were the majority. The Uncompahgre Plateau site had equal proportions of the T and C chemotypes and thus the chances were equal that the historic sample would match one of these. After excluding both the Boulder Canyon herbarium sample and the Uncompahgre Plateau site, the chemotypes from 15 of the 18 historic herbarium samples matched those of the most abundant chemotypes found after recent sampling, which did not differ from expected [χ^2^ (1, *N* = 18) = 0.114, *p* = .735].

**FIGURE 6:**
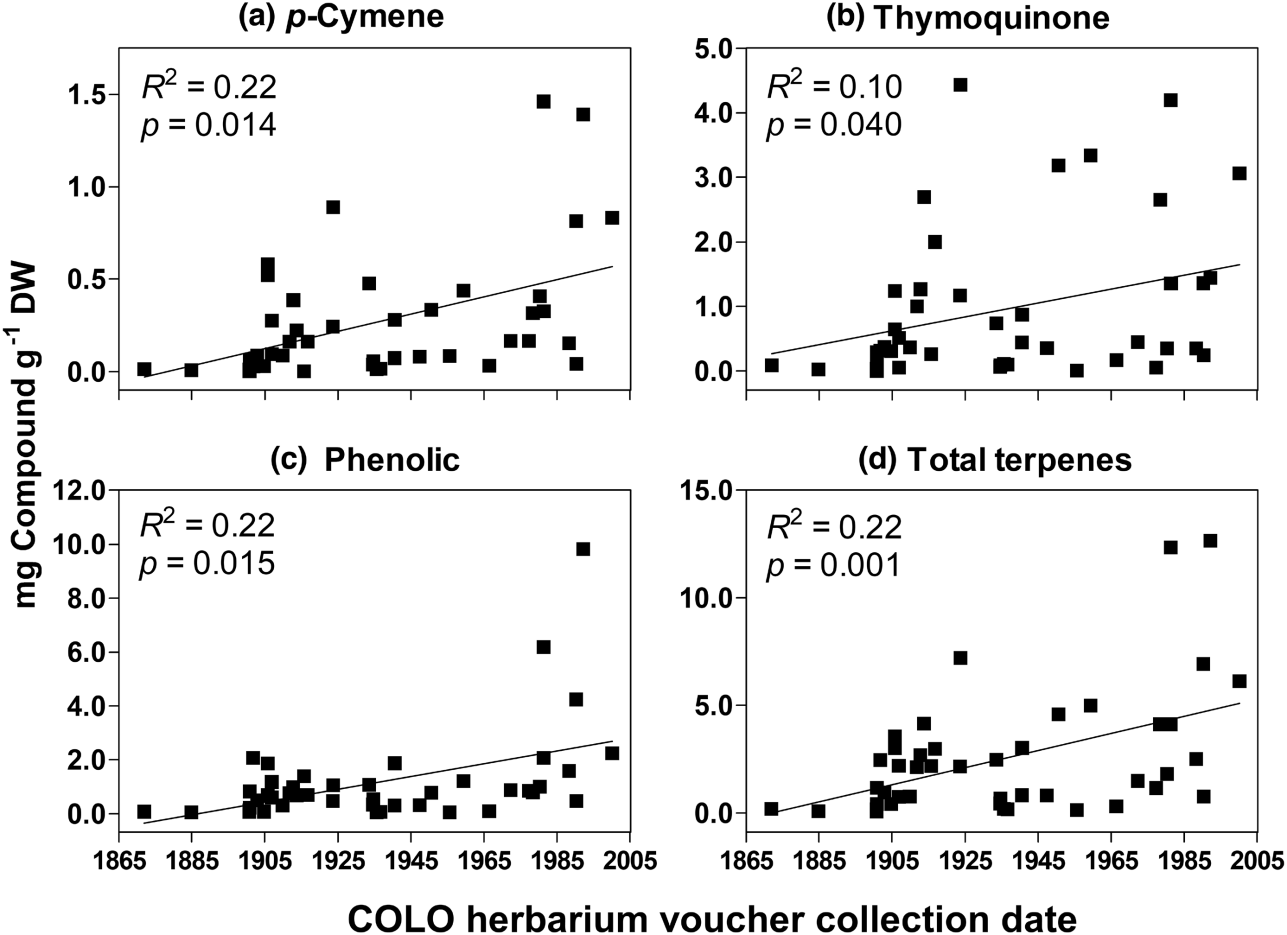
The major terpenes detected in 44 accessions of *Monarda fistulosa* from the University of Colorado Museum Herbarium (COLO). Regression results of the content [mg/g dry weight (DW)] of the main terpenes versus collection date.

Regression analyses of the amounts of compounds over time (collection date) showed positive relationships for the content of *p*‐cymene (Figure 6a), thymoquinone (Figure 6b), total phenolic monoterpenes (Figure 6c), and total terpenes (Figure 6d). Thus, the amounts of all compounds measured and the total decreased with herbarium sample age.

## 4 DISCUSSION

### 4.1 Spatial variation of *M. fistulosa* chemistry

The results of this study revealed that even within a restricted part of its range a species can have considerable amounts of secondary chemistry variation at different spatial and temporal scales. The broadest geographic scale showed that most of the *M. fistulosa* populations analyzed contained plants of either T or C chemotypes; however, the ratio of these two chemotypes varied across the landscape, even over relatively short distances as seen in the intense sampling in Colorado. While the reasons for the chemotype patterns observed in *M. fistulosa* are not immediately clear, abiotic selective forces do not seem to be important. A regression analysis of *M. fistulosa* chemotype percentages (%T) versus elevation, a proxy of temperature, of the Colorado sites was not significant (*R* ^2^ = 0.01, *p* = .43). Studies of other labiates that also have T and C chemotypes have been mixed in their explanation of geographic patterns due to abiotic parameters. Work with *Origanum vulgare* ssp. *hirtum* in Greece revealed that although the total amount of essential oil in plants of both T and C chemotypes could be partially explained by elevation, summer water deficiency, and thermal efficiency; the spatial distribution was not correlated with any of these factors (Vokou et al., 1993). By contrast, in reciprocal transplant experiments of *Thymus vulgaris* in southern France, Thompson et al. (2007) found that not only are T and C plants restricted to warmer areas as compared to nonphenolic chemotypes plants (including G and L), but T plants were better able to resist early freezing episodes than C plants. While the differential response to cold in thyme phenolic chemotypes helps determine where they are found, these abiotic effects probably matter less to *M. fistulosa*, due to a different growth strategy. Although both thyme and *M. fistulosa* are perennials, most thyme species have above‐ground foliage year‐round, including during periods when it would be exposed to extreme cold. By contrast, *M. fistulosa* plants die back completely with the onset of winter and then resprout from roots the next spring.

Biotic factors may be more important in determining chemical patterns in *M. fistulosa*. For instance, recent work with *M. fistulosa* populations in Montana and Wisconsin showed that important ecological traits, such as terpene production versus plant biomass (growth‐defense tradeoff), can vary within and among populations in different regions (Hahn et al., 2021). Also, while T and C chemotypes seem to be more resistant overall to herbivores than other chemotypes (Linhart & Thompson, 1999), the *M. fistulosa* specialist tortoise beetle *Physonota unipunctata* performed better on and did more damage to T plants (Keefover‐Ring, 2015), which could shift population proportions of these two chemotypes. Finally, genetic dominance of specific chemotypes could also alter population chemistry. In *T. vulgaris*, monoterpene chemotypes are under the control of an epistatic cascade, where geraniol plants are dominant over all other chemotypes and thymol plants are completely recessive (Vernet et al., 1986). *T. vulgaris* also contains the same four chemotypes that I found in *M. fistulosa* with the order of dominance determined to be: G > L > C > T. This pattern also seems to be the case in the genus *Monarda*, at least for G, L, and T individuals (Marshall & Scora, 1972). Thus, pollination and seed dispersal would be biotic factors important in shaping *M. fistulosa* chemotype configurations.

The smaller spatial scale of different parts of a single plant also showed considerable amounts of chemical variation in *M. fistulosa*, with differing patterns among compounds. Stems and roots were the least chemically defended in both the C and G plants tested, a pattern seen in other mint family species (Tajbakhsh et al., 2007; Velickovic et al., 2002). These structures may depend primarily upon their woodiness for protection. Compared with stems and roots, the other tissues of *M. fistulosa* analyzed had relatively high amounts of terpenes, and in general, most compounds had similar levels in reproductive parts (flower petals and calyces) and leaves, with bracts usually lower. The relatively high amounts of terpenoids in the reproductive parts of *M. fistulosa* fits with optimal defense theory, which predicts that plant tissues most important to fitness should be most constitutively defended (McKey, 1974). However, higher levels of secondary compounds in floral parts can also be a liability when specialist herbivores are concerned. Smallegange et al. (2007) found that although the flowers of *Brassica nigra* contained levels of glucosinolates up to five times higher than those of leaves, they sustained much more damage from specialist pierid caterpillars. *M. fistulosa* may suffer the same fate in populations where both larvae and adults of the one‐spotted tortoise beetle *Physonota unipunctata* feed on foliage and floral parts, causing significant reductions in seed set due to feeding on terpene‐rich flower heads (Keefover‐Ring, 2015).

### 4.2 Temporal variation of *M. fistulosa* chemistry

The short‐term temporal changes in *M. fistulosa* chemistry seen during the 2007 growing season were dramatic and T and C chemotypes demonstrated somewhat different patterns in both their individual terpenes and total compounds. In general, essential oils in both chemotypes were relatively low early in the spring, reached their highest levels in late summer during plant flowering, and then steadily declined to low final levels after leaf senescence. The most notable differences among chemotypes were seen in samples collected on June 1 that showed T plants had significantly higher amounts of total terpenes, due mostly to a much higher amount of the phenolic precursor γ‐terpinene. While the chemical defense of labiates is usually thought to be constitutive, these results show that the chemical phenotype of these plants is quite variable during a single season and this may present a “moving target” to potential herbivores (Adler & Karban, 1994), or even to pollinators, since the changing amounts of the more volatile monoterpenes (γ‐terpinene and *p*‐cymene) in plant tissues during the season will lead to temporal changes in terpene emissions (Keefover‐Ring, 2013). Finally, the seasonal change in essential oil composition also indicates that the allelopathic potential of *M. fistulosa* may change over the growing season (Linhart et al., 2015).

Comparisons of *M. fistulosa* herbarium samples with current populations and the detection of new chemotypes are both indicators of the possible long‐term structure of the species' chemistry. First, due to their inherently stable aromatic structure and very high boiling points for monoterpenes (thymol, 232°C, and carvacrol, 238°C), the chemotypes of dried T and C plants should be preserved for long periods of time. My own recent testing of dried *M. fistulosa* foliage collected 16–18 years ago for this work showed that chemotypes had not changed. Overall, results from the herbarium samples indicated that *M. fistulosa* chemotype distributions may have remained stable for more than a century in Colorado, with the chemotype identities of most historic samples matching those of contemporary populations. Although a single plant sample from each location represents only a snapshot of a population's past chemical diversity; the frequency at which the past and present samples agreed provides some evidence of little change in chemotypes. This should not be surprising, since even the time frame of 125 years is relatively short to expect evolutionary change in a perennial plant.

While material from herbarium samples is routinely used for molecular genetic analysis, this resource has less often been used to assess the past chemical diversity of a species (Almasirad et al., 2007; Baser et al., 2005; Novak et al., 2002), and only rarely in an ecological context (Berenbaum & Zangerl, 1998; Zangerl & Berenbaum, 2005). Zangrel and Berenbaum (2005) used herbarium specimens of wild parsnip (*Pastinaca sativa*) to see whether close association with a specialized lepidopteron herbivore caused changes in furanocoumarin amounts compared with contemporary plants. In their study, even the oldest plants contained furanocoumarins, which also decreased in amount with sample age. The historic *M. fistulosa* samples I tested demonstrated that essential oils can be recovered from plants collected up to 125 years ago and that chemotypes of even the oldest individuals could be determined since the dominant terpene of T and C chemotypes of *M*. *fistulosa* could still be detected. However, herbarium samples will lose even these less volatile terpenes (thymol and carvacrol) over time, as can be seen from the regressions of compound content versus sample collection year. In addition, differential loss of terpenes due to very dissimilar volatilities (Keefover‐Ring, 2013), combined with compound degradation, such as the conversion of thymol and carvacrol to thymoquinone (Jukic & Milos, 2005; Krause et al., 2021), led to results that cannot be directly compared with the exact chemical profiles of contemporary plants. Thus, due to the physical properties of mono‐ and sesquiterpenes, herbarium samples containing essential oils can probably only be used in qualitative comparisons to contemporary material (Novak et al., 2002).

The appearance of a new trait in a population may lead to evolution in that species if that trait possesses some advantage over an existing one. In the case of essential oil phenotypes in *M. fistulosa*, for a large extent of its range, the T and C chemotypes have been very successful in remaining the only plant chemotypes, possibly due to the greater toxicity these compounds have shown toward a range of herbivores and parasites (Linhart & Thompson, 1995, 1999), and plant competitors (Linhart et al., 2015; Tarayre et al., 1995), compared with other monoterpenes. However, two other chemotypes of *M. fistulosa* have arisen—G chemotype plants in Canada and L chemotype plants in Colorado. Marshall and Scora (1972) were the first to formally document the existence of a geraniol chemotype of wild bergamot in and around Spruce Woods Provincial Park, known to exist since at least the mid‐1950s. They theorized that the G chemotype probably represents a recent mutation in the species, since it is very restricted in its distribution, but can still readily interbreed with other *M. fistulosa* chemotypes (Marshall & Scora, 1972). My analyses of *M. fistulosa* populations in Manitoba more than 30 years after their collections show that this new chemotype has persisted, although without detailed baseline population chemotype demographics it is difficult to tell if the proportions of G to T and C chemotypes have changed over time. At the opposite latitude of my sampling area in southern Colorado, another new *M. fistulosa* chemotype was discovered with plants containing (*R*)‐(−)‐linalool as their dominant monoterpene. All other plants at the site were either C or T chemotype plants and there appeared to be no morphological differences between these and the L individuals. Only three plants of this new chemotype were found, out of the almost 600 assayed by chemical or olfactory means (L plants smell very different than T or C) at this site, thus, it seems likely that these plants may also represent a very recent mutation (Marshall & Scora, 1972). Unlike the new chemical race of geraniol plants in Canada, the fate of this new chemotype remains to be seen, although they were still at the site in the summer of 2019 (Keefover‐Ring, personal observation). The process of evolution is a series of trials with new phenotypes, and sometimes these experiments fail. While evidence from other labiates shows that T and C chemotypes certainly have an advantage over nonphenolics with respect to withstanding herbivory (Linhart & Thompson, 1999) or inhibiting competitors (Linhart et al., 2015), G and L chemotypes may possess characteristics that may favor them. For instance, both of these chemotypes produce monoterpenes (linalool and geraniol) known to attract pollinators (Andersson & Dobson, 2003; Schmidt, 1999) or deter herbivores, such as grasshoppers (Linhart & Thompson, 1999) or aphids (Linhart et al., 2005). Also, data from Marshall and Scora (1972) hinted that the inheritance pattern of *M. fistulosa* chemotypes may be G > L > C > T, as is the case in *T. vulgaris* (Vernet et al., 1986). If so, it is likely that the new chemotypes will continue to expand their range since any pollen transfer from G and L plants to C or T plants will mostly result in G or L offspring (Marshall & Scora, 1972; Vernet et al., 1986). Recent evidence shows that *M. fistulosa* individuals with chemotypes other than T or C may be more widespread than previously thought. In the summers of 2020 and 2021, I detected a few G and L plants in remnant or restored prairie sites near Madison, WI (Keefover‐Ring, personal observation). In addition, at one of these sites, several plants were found containing 1,8‐cineole (eucalyptol; scent of eucalyptus) and α‐terpineol (pine‐like scent), which alternated as the largest peak in their essential oil profile (Keefover‐Ring, personal observation). While these chemotypes have never been described in *M. fistulosa*, Scora (1967) found relatively high levels of both of these monoterpenes in different *M. fistulosa* varieties. In addition, *T. vulgaris* has both 1,8‐cineole and α‐terpineol chemotypes in some populations (Keefover‐Ring et al., 2009), increasing the number of chemotypes shared by these two mint species, despite their geographic separation. The chemical characterization of these Wisconsin sites is ongoing, although the majority of plants appear to be mostly T or C chemotypes (Keefover‐Ring, personal observation).

## 5 CONCLUSIONS

*M. fistulosa* demonstrated large amounts of chemical diversity at all of the spatial and temporal scales examined. At the landscape level, the T and C chemotypes were the most widespread and the concentrations of their main monoterpene explained most of the variation in chemistry across populations. While this species is dominated by these two main chemotypes, the persistence of a recent G chemotype in southern Manitoba and the discovery of a new L chemotype in southern Colorado highlight the possibility that the chemotype distributions of *M. fistulosa* could change, which will influence chemistry‐driven interactions between this plant species and other community members. The essential oil variation within a plant, among different parts, revealed that foliage and reproductive structures are well defended, especially compared with stems and roots. Furthermore, many of the individual terpenes expressed separate patterns in different plant tissues. Temporal variation in *M. fistulosa*, at the scale of a single season, showed that plants start relatively low in total terpenes, reach a peak in late summer, and decrease sharply as leaves senesce. In addition, separate main monoterpenes follow different seasonal trajectories, and in some cases, the behavior of the same compound diverges among chemotypes. Results of analyses of herbarium samples with contemporary material seemed to indicate that the chemotype distributions of *M. fistulosa* have for the most part been stable for at least the last century. All of these patterns of secondary chemistry variation in *M. fistulosa* have implications for the chemical ecology of the species, possibly leading to different interactions with other community members over both space and time.

## AUTHOR CONTRIBUTIONS

**Ken Keefover‐Ring:** Conceptualization (equal); data curation (equal); formal analysis (equal); funding acquisition (equal); investigation (equal); methodology (equal); project administration (equal); writing – original draft (equal); writing – review and editing (equal).

## FUNDING INFORMATION

This work was supported by grants from the University of Colorado Museum Walker Van Riper Fund, the Colorado Native Plant Society, and a 2020 Fall Competition Grant from the University of Wisconsin‐Madison, Office of the Vice Chancellor for Research, and Graduate Education with funding from the Wisconsin Alumni Research Foundation to K.K.‐R.

## CONFLICT OF INTEREST

None declared.

## Data Availability

PCA site comparison and chemistry data: Dryad DOI https://doi.org/10.5061/dryad.18931zd12.

## APPENDIX A

The chemical biogeography of a widespread aromatic plant species shows both spatial and temporal variation

See Tables A1 and A2

**TABLE A1:** GPS coordinates, chemotype counts, and collection date of *Monarda fistulosa* leaf collection sites for chemotype mapping

Sites	GPS coordinates		Chemotype				Collection date
	N	W	C	T	G	L	
*Canada*							
Aweme	49.70846	99.60201	1	13	0	0	26‐Jul‐04
Manitoba‐1	49.88105	99.16388	16	0	0	0	25‐Jul‐04
Manitoba‐2	49.90125	99.11055	22	0	3	0	25‐Jul‐04
Manitoba‐3	49.90519	99.13282	8	0	0	0	25‐Jul‐04
Manitoba‐4	49.90967	99.13271	19	0	3	0	25‐Jul‐04
Manitoba‐5	49.95134	98.92655	17	0	1	0	26‐Jul‐04
Manitoba‐6	49.64935	99.5523	1	6	0	0	26‐Jul‐04
Spruce Woods‐1	49.70923	99.26476	19	1	0	0	24‐Jul‐04
Spruce Woods‐2	49.71694	99.27588	10	0	0	0	24‐Jul‐04
Spruce Woods‐5	49.71828	99.28900	20	2	0	0	24‐Jul‐04
Spruce Woods‐6	49.89442	99.52022	20	0	0	0	25‐Jul‐04
Spruce Woods‐7	49.88808	99.51678	19	1	0	0	25‐Jul‐04
Spruce Woods‐8	49.70009	99.25545	16	0	9	0	27‐Jul‐04
Spruce Woods‐9	49.65536	99.24690	11	1	1	0	27‐Jul‐04
*N. and S. Dakota and Wyoming*							
Carpio, ND	48.43685	101.7258	0	16	0	0	29‐Jul‐04
New Town, ND	47.96736	102.7285	6	15	0	0	29‐Jul‐04
Roosevelt Expressway, ND	47.54307	103.2372	14	1	0	0	29‐Jul‐04
Black Hills, WY	43.93157	104.1841	11	4	0	0	30‐Jul‐04
Black Hills‐1, SD	44.29551	103.8826	0	14	0	0	30‐Jul‐04
Black Hills‐2, SD	44.17639	104.0507	14	0	0	0	30‐Jul‐04
*Boulder County, CO*							
Armstrong	39.96289	105.3137	18	12	0	0	15‐Jul‐05
Walker Ranch‐1	39.94851	105.3381	0	10	0	0	23‐Aug‐02
Walker Ranch‐2	39.94427	105.3370	0	10	0	0	23‐Aug‐02
Walker Ranch‐3	39.94257	105.3382	1	10	0	0	23‐Aug‐02
Walker Ranch‐4	39.94066	105.3407	0	10	0	0	23‐Aug‐02
Walker Ranch‐5	39.93841	105.3405	10	0	0	0	23‐Aug‐02
Walker Ranch‐6	39.93452	105.3385	0	10	0	0	23‐Aug‐02
Walker Ranch‐7	39.93429	105.3382	0	10	0	0	23‐Aug‐02
Walker Ranch‐8	39.92997	105.3358	0	10	0	0	23‐Aug‐02
Walker Ranch‐9	39.94125	105.3135	7	3	0	0	23‐Aug‐02
Walker Ranch‐10	39.94224	105.3144	3	7	0	0	23‐Aug‐02
Walker Ranch‐11	39.94662	105.3172	0	10	0	0	23‐Aug‐02
Walker Ranch‐12	39.94965	105.3186	2	8	0	0	23‐Aug‐02
Walker Ranch‐13	39.95164	105.3194	6	4	0	0	23‐Aug‐02
Walker Ranch‐14	39.95283	105.3206	3	7	0	0	23‐Aug‐02
Walker Ranch‐15	39.94953	105.3360	4	6	0	0	23‐Aug‐02
Dowdy Draw	39.91794	105.2591	26	0	0	0	20‐Jul‐03
Mount Sanitas	40.02074	105.2965	7	11	0	0	20‐Jul‐03
Mesa Trail	39.94261	105.2630	1	0	0	0	25‐May‐03
Boulder Canyon	39.98234	105.4449	0	16	0	0	7‐Jul‐03
Left Hand Canyon‐1	40.13232	105.2934	12	13	0	0	7‐Jul‐03
Left Hand Canyon‐2	40.10455	105.3376	20	10	0	0	7‐Jul‐03
Left Hand Canyon‐3	40.13915	105.4147	6	23	0	0	7‐Jul‐03
Magnolia	39.97750	105.3987	1	19	0	0	7‐Jul‐03
Boulder Can Public Service	40.00718	105.3295	0	6	0	0	7‐Jul‐03
Four Mile Canyon‐1	40.04766	105.3674	9	8	0	0	9‐Jul‐03
Four Mile Canyon‐2	40.02952	105.3410	0	2	0	0	9‐Jul‐03
Salina‐02	40.05147	105.3726	0	1	0	0	9‐Jul‐03
Salina‐01	40.05422	105.3778	0	2	0	0	9‐Jul‐03
Gregory Canyon Trailhead	39.99740	105.2930	6	25	0	0	10‐Jul‐03
Shanahan Ridge	39.96173	105.2633	17	0	0	0	17‐Jul‐03
Rocky Flats	39.91309	105.1845	8	0	0	0	20‐Jul‐03
Bear Canyon‐1	39.97388	105.2826	16	4	0	0	21‐Jul‐03
Bear Canyon‐2	39.97811	105.2836	20	0	0	0	21‐Jul‐03
Homestead Trail	39.94388	105.2754	21	0	0	0	6‐Aug‐03
Shadow Canyon	39.94347	105.2792	21	0	0	0	6‐Aug‐03
Upper Gregory Canyon	39.99758	105.3066	19	103	0	0	13‐Jul‐05
Eldora	39.94432	105.5598	22	3	0	0	24‐Jul‐05
Sugar Loaf	40.02260	105.4440	4	26	0	0	24‐Jul‐05
County Road 103	39.99699	105.5027	11	20	0	0	24‐Jul‐05
Sugar Loaf Rd	40.00190	105.4729	0	20	0	0	24‐Jul‐05
*Colorado (w/o Boulder County)*							
Frazer Meadow Trail‐1	39.83889	105.4099	8	18	0	0	24‐Aug‐03
Frazer Meadow Trail‐2	39.83372	105.4090	10	11	0	0	24‐Aug‐03
Coal Creek Canyon Rd‐1	39.87948	105.3189	2	13	0	0	18‐Jul‐03
Coal Creek Canyon Rd‐2	39.87869	105.2693	10	0	0	0	24‐Aug‐02
Estes Park	40.32921	105.5735	0	10	0	0	18‐Jul‐02
Pinewood	40.27171	105.3567	10	0	0	0	18‐Jul‐02
North Platte River Rd	39.34364	105.1773	0	10	0	0	9‐Aug‐02
Mount Gilbrath Park	39.77247	105.2544	25	3	0	0	11‐Jul‐03
Golden Gate Rd	39.76789	105.2778	29	1	0	0	11‐Jul‐03
Mount Vernon	39.71960	105.2881	14	0	0	0	11‐Jul‐03
Crescent Meadows	39.92902	105.3405	37	7	0	0	16‐Jul‐03
Horsetooth Mtn Park‐1	40.52849	105.1816	2	18	0	0	22‐Jul‐03
Horsetooth Mtn Park‐2	40.53105	105.1877	1	19	0	0	22‐Jul‐03
Masonville	40.56683	105.3123	11	9	0	0	22‐Jul‐03
Masonville Rd	40.67073	105.3833	2	18	0	0	22‐Jul‐03
Poudre Canyon‐1	40.69104	105.4882	0	20	0	0	23‐Jul‐03
Poudre Canyon‐2	40.68257	105.4078	0	20	0	0	23‐Jul‐03
Poudre Canyon‐3	40.69145	105.2543	0	20	0	0	23‐Jul‐03
Palmer Lake	39.12746	104.9071	13	1	0	0	24‐Jul‐03
Highway 12	37.25548	105.0368	23	12	0	3	8‐Jul‐05
East Canyon Creek	39.62123	107.4180	0	7	0	0	14‐Aug‐03
Aldrich Lakes	40.17511	107.6844	29	0	0	0	15‐Aug‐03
Uncompadre Plateau	38.54447	108.4356	15	15	0	0	15‐Aug‐03
Maroon Bells	39.22863	107.1944	2	15	0	0	23‐Sep‐05
Gleneagle	39.04536	104.8443	5	14	0	0	24‐Nov‐05

*Note*: C = carvacrol, T = thymol, G = geraniol, and L = (*R*)‐(−)‐linalool. See Figure 2 in the main text.

**TABLE A2:** University of Colorado Museum Herbarium (COLO) *Monarda fistulosa* samples analyzed for terpene chemotype and chemotype ratios from 20 of the sites recently revisited.

Site (Colorado county) COLO accession #	Historic collection date	Historic chemotype	Recent chemotypes	Recent collection date
Glen Eyrie, Colorado Springs (El Paso) 101490	Jul 17, 1872	T	N/A	N/A
E. Denver along the Platte (Unknown) 23474	Jul 1885	C	N/A	N/A
Buffalo Park (Unknown) 23479	Jul 11, 1901	C	N/A	N/A
Palmer Lake (El Paso) 23483	Jul 13, 1901	C	13 C: 1 T	Jul 24, 2003
Gregory Canyon, near Boulder (Boulder) 23470	Aug 12, 1901	T	6 C: 25 T	Jul 17, 2003
Salina (Boulder) 23456	Aug 20, 1901	T	0 C: 3 T	Jul 9, 2003
Manitou (El Paso) 23482	Jul 12, 1902	T	N/A	N/A
Miller Gulch, Buffalo Park (Unknown) 23484	Sep 7, 1903	T	N/A	N/A
Below brickyard, near Boulder (Boulder) 55139	Jul 18, 1905	T	N/A	N/A
Bear Canyon (Boulder) 23459	Jul 16, 1906	C	16 C: 4 T	Jul 21, 2003
Eldora town site (Boulder) 23468	Jul 28, 1906	C	22 C: 3 T	Jul 24, 2005
Magnolia (Boulder) 23485	Aug 22, 1907	T	1 C: 19 T	Jul 7, 2003
South Boulder Canyon (Boulder) 23454	Aug 24, 1907	C	N/A	N/A
Meeker (Rio Blanco) 23487	Aug 9, 1910	T	N/A	N/A
Naturita (Montrose) 332651 23473	Aug 1, 1912 Jul 17, 1914	T T	N/A	N/A
Tabeguache Basin (Montrose) 23457	Aug 19, 1913	C	N/A	N/A
Berkeley (Arapahoe) 23476	Jul 16, 1916	T	N/A	N/A
Poudre Canon (Larimer) 322192	Jul 25, 1917	T	0 C: 20 T 0 C: 20 T 0 C: 20 T	Jul 22, 2003
Along Piedra River (Archuleta) 23448	Jul 8, 1924	C	N/A	N/A
Near Boulder (Boulder) 23478	Jul 20, 1924	C	N/A	N/A
Cuchara Valley (Huerfano) 23451	Jul 6, 1934	T	10 C: 9 T	Jul 25, 2003
Aldrich Lake (Rio Blanco) 23489, 23493	Jul 15, 1935 Jul 22, 1937	C C	29 C: 0 T	Aug 14, 2003
Mouth of Left Hand Canyon (Boulder) 23494	Aug 7, 1935	C	13 C: 12 T	Jul 7, 2003
Denver Mountain Parks near Golden (Jefferson) 23491	Jul 5, 1941	C	19 C: 2 T	Jul 11, 2003
Mt. Vernon Country Club (Jefferson) 23490	Aug 3, 1941	C	14 C: 0 T	Jul 11, 2003
Atop W. Mesa de Maya (Las Animas) 56072	Jun 23, 1948	T	N/A	N/A
North Thompson Creek Canyon (Pitkin) 64455	Jul 28, 1951	T	N/A	N/A
Estes Park, Rocky Mtn. Natl. Park (Larimer) 123392	Aug 28, 1956	C	0 C: 10 T	2002
3 miles north of Arboles (Archuleta) 215235	Jul 22, 1960	T	N/A	N/A
Boulder Canyon at Public Service (Boulder) 214765	Jul 30, 1967	T/C	0 C: 6 T	Jul 7, 2003
Rocky Flats site (Jefferson) 274751	Jul 9, 1973	C	8 C: 0 T	Jul 20, 2003
Along Dolores River (Montezuma) 431247	Summer 1978	C	N/A	N/A
Lost Creek Area, Tarryall Mtns (Jefferson) 429154	Aug 8, 1979	T	N/A	N/A
Spring Creek, W of Bull Creek (Mesa) 382665	Jul 11, 1981	C	N/A	N/A
Uncompahgre Plateau (Mesa) 383696	Jul 19, 1982	T	15 C: 15 T	Aug 15, 2003
Weminuche Wilderness (La Plata) 385607	Aug 23, 1982	C	N/A	N/A
Black Forest Along Kettle Creek (El Paso) 494842	Jul 24, 1989	C	N/A	N/A
Flat Tops/White River Plateau (Garfield) 466589	Jul 25, 1990	C	0 C: 7 T	Jul 14, 2003
Boulder Mt Parks West side of Bear Pk & S Boulder Pk (Boulder) 450606	Jul 19, 1991	T	N/A	N/A
South Table Mt (Jefferson) 449215	Aug 19, 1991	C	N/A	N/A
South Boulder Foothills Shanahan (Boulder) 453922	Jun 28, 1993	C	16 C: 0 T	Jul 17, 2003
Big Elk Meadows Road (Larimer) 521233	Jul 10, 2001	T	N/A	N/A
Platte Cañon (Unknown) 101517	25 May no year	C	N/A	N/A

Abbreviations: C, carvacrol; T, thymol.
